# Dietary Addition of Tributyrin Improved the Production Performance, Antioxidant Ability and Intestinal Health in Weaned Rabbits

**DOI:** 10.3390/ani15131923

**Published:** 2025-06-29

**Authors:** Nanbin Zhang, Xianghui Li, Huijie Xu, Fuchang Li, Lei Liu

**Affiliations:** Key Laboratory of Efficient Utilization of Non-Grain Feed Resources (Co-Construction by Ministry and Province), Ministry of Agriculture and Rural Affairs, Shandong Provincial Key Laboratory of Animal Nutrition and Efficient Feeding, Department of Animal Science and Technology, Shandong Agricultural University, Taian 271017, China; zhangnb@haid.com.cn (N.Z.); 15753808707@163.com (X.L.); p364589xu@163.com (H.X.); chlf@sdau.edu.cn (F.L.)

**Keywords:** tributyrin, production performance, antioxidant, intestinal health, rabbit

## Abstract

The present study was conducted to investigate the effects of dietary addition of tributyrin on growth performance, antioxidant and intestinal health in weaned rabbits. Weaned Hyla rabbits (35 d, *n* = 1280) of similar body weight were randomly divided into four groups (eight replicates per group, 40 rabbits per replicate), fed diets with 0, 0.1%, 0.2% or 0.4% tributyrin addition. A 3-day pre-experimental period was followed by a 36-day trial period. Our results show that dietary addition of 0.2% tributyrin significantly increased the average daily gain (ADG) and final body weight (FBW) of rabbits, and decreased the diarrhea rate (*p* < 0.05). Dietary addition of 0.1% and 0.2% tributyrin significantly increased the average carcass weight and liver weight (*p* < 0.05). In the liver, dietary addition of tributyrin increased the content of glutathione peroxidase (GSH-PX) and total superoxide dismutase (T-SOD) compared with the control group (*p* < 0.05). Dietary addition of 0.1% and 0.2% tributyrin significantly increased the serum GSH-PX level (*p* < 0.05). Dietary addition of 0.2% tributyrin significantly increased the serum T-SOD level (*p* < 0.05). Dietary addition of 0.2% tributyrin significantly increased villus height/crypt depth (V/C) in the duodenum (*p* < 0.05). Dietary addition of 0.1–0.4% tributyrin significantly increased V/C in the jejunum (*p* < 0.05). Dietary addition of 0.1–0.2% tributyrin significantly increased the V/C in the ileum, but significantly decreased the content of serum diamine oxidase (DAO) and of D-lactic acid in serum (*p* < 0.05). In conclusion, dietary addition of tributyrin can improve the production performance and antioxidant ability of liver and serum, and improve the intestinal health by decreasing the diarrhea rate and the intestinal permeability and improving intestinal morphology in weaned rabbits. Based on the present results, the optimal level of tributyrin is 0.2% in the weaned rabbit diet.

## Simple Summary

Post-weaning, rabbits often experience digestive tract problems, resulting in growth retardation or diarrhea infectious diseases. In the present study, we evaluated the application effect of tributyrin in weaned rabbit diets. The results show that the addition of 0.2% tributyrin in the diet improved the production performance and antioxidant ability of weaned meat rabbits, decreased the diarrhea rate and the intestinal permeability and improved intestinal morphology. These findings propose tributyrin as a protective agent for intestinal health to use in commercial rabbit production.

## 1. Introduction

Weanling stress rabbits can induce gut microbiota dysbiosis, intestinal damage, oxidative stress or inflammation, resulting in their weakened digestion and absorption capacity, growth retardation or frequently diarrhea infectious diseases [1,2]. Antibiotics have been overused to control mortality, burdening the environment and public health. Currently, the identification and evaluation of safe and effective antibiotic alternatives or products that reduce the application of antibiotics are urgent and important for maintaining intestinal health and production efficiency in intensive rabbit farming systems. Some natural, eco-friendly, non-antibiotic feed supplements are being applied in rabbit diets to maintain intestinal health. For instance, oregano essential oil has been shown to enhance growth performance, feed efficiency and antioxidant activity in rabbits [3]. It has been reported that dietary butyrate supplementation has a positive effect on growth performance, the efficiency of nutrient digestion and health [4]. Butyrate alleviating diarrhea and some intestinal diseases is well-documented in numerous studies. Mathew et al. demonstrated that butyrate exhibits antioxidant effects by increasing glutathione peroxidase (GSH-PX) level and anti-inflammatory response by activating nuclear factor kappa-B (NF-κB) signal [5]. Additionally, butyrate protects the gut lining and enhances intestinal barrier integrity by up-regulating tight junction protein expressions. It also reduces inflammation through the modulation of cytokine responses in weaned piglets [6]. However, butyrate may be challenging because of the short half-life in plasma and unpleasant smell [7]. Tributyrin does not have the butyrate-distinctive offensive odor and is made up of three molecules of butyric acid, which contributes to its longer and sustained effect on gut cells and more favorable pharmacokinetics [8]. Tributyrin is present in the milk of females. Young animals (e.g., rabbits, piglets and calves) learn this taste and later prefer it as the so-called taste of childhood. So, adding tributyrin in the diet increases the palatability of the feed [9].

The previous study shows that tributyrin can decrease inflammatory factors and alleviate heat stress in cows [10]. Dietary tributyrin addition can ameliorate oxidative stress, alleviate the inflammation and maintain intestinal integrity in piglets [11]. Tributyrin can reduce the occurrence of diseases caused by pathogens, reduce oxidative damage and improve the intestinal barrier function of broilers. So tributyrin is widely applied in the diets of weaning piglets [12] and broilers [13]. The application effect or application dosage of tributyrin remains unclear in rabbits. Weaned rabbits experience more digestive tract problems than weaned piglets and broilers. The intestinal wall of rabbits is thinner than other livestock (e.g., broilers), which increases intestinal permeability. Due to coprophagy, rabbits are prone to ingesting some pathogenic microorganisms, which leads to frequent outbreaks of intestinal problems. High diarrhea rate or low productivity effects caused great economic losses in growing rabbits. The present study was conducted to investigate the effects of dietary addition of different levels of tributyrin on production performance, antioxidant ability and intestinal health in rabbits.

## 2. Materials and Methods

### 2.1. Experimental Protocol and Sample Collection

At 35 days of age, 1280 weaned Hyla rabbits (male-female ratio of 1:1) with similar body weights (1.04 ± 0.01 kg) were randomly divided into 4 treatment groups (8 replicates per group, 40 rabbits per replicate). The rabbits were fed a basic diet (control group) or fed the experimental diet supplemented with 0.1%, 0.2% or 0.4% of tributyrin (Liaocheng Jiashi Co., Ltd., Liaocheng, China). Rabbits were individually housed in custom-built plastic cages (60 × 40 × 40 cm). A 3-day pre-experimental period was followed by a 36-day trial period. Throughout the experiment, animals were fed twice daily (08:00 and 16:00) with a steam-pelleted (4 mm diameter) diet. The ingredients and chemical composition of the basal diet are listed in Table 1. With unlimited access to food and water, the rabbits were given incrementally increasing daily feed amounts. Their housing conditions were maintained at 20–23 °C under a 12-h light/dark cycle. The study was conducted in Shandong Huifu Agriculture and Animal Husbandry Development Co., Ltd. (Liaocheng, China).

At the end of the trial, 8 rabbits from every treatment (1 rabbit per replicate) were selected for 10 mL cardiac blood collection, after being placed for 30 min centrifuged by using centrifuge (centrifuged at 3000 r/min for 10 min). Then, the liquid supernatant was drawn and transferred to a 1.5 mL centrifuge tube using a pipette, and stored in a refrigerator at −20 °C. The rabbits were electrically stunned and slaughtered by exsanguination. Then half-bore weight and full-bore weight were immediately measured. The liver was removed from each rabbit, and the liver samples were collected and cut. Then, the samples were transferred to a 1 mL centrifuge tube with normal saline for homogenization by using a homogenizer, centrifuged (centrifuged at 2000 r/min for 8 min), and the supernatant liquid was collected. The whole intestine was carefully removed from each rabbit, and 2 cm segments from the duodenum, jejunum and ileum were collected. Then, the segments were flushed gently with ice-cold phosphate-buffered saline (PBS, pH 7.4), rapidly frozen in liquid nitrogen and stored at an ultra-low temperature freezer (−80 °C) until analysis.

### 2.2. Methods

#### 2.2.1. Growth Performance

The initial body weight (IBW) and final body weight (FBW) of individual rabbits were recorded. Average daily gain (ADG), average daily feed intake (ADFI) and feed gain ratio (F/G) of each replicate were calculated. ADFI = total feed intake/test days. ADG = (terminal weight − initial weight)/test days. F/G = ADFI/ADG.

#### 2.2.2. Analysis of Slaughter Performance

Before slaughter, the test rabbits were weighed on an empty stomach and their pre-slaughter live weights were recorded. After slaughter, the rabbits were eviscerated by removing the intestines, head, stomach, fur, lungs, esophagus, trachea, spleen, pancreas and reproductive organs, leaving only the heart, liver and kidneys. The weight at this stage was recorded as the half-bore weight. Subsequently, the remaining organs (heart, liver and kidneys) were also excised, and the carcass was weighed once more to determine the full-bore weight. The half-bore rate, full-bore rate, spleen index and liver index were calculated.

Half-bore rate = half-bore weight/pre-slaughter live weight.

Full-bore rate = full-bore weight/pre-slaughter live weight.

Spleen index = spleen weight/pre-slaughter live weight.

Liver index = liver weight/pre-slaughter live weight.

#### 2.2.3. Analysis of Meat Quality

Samples of the thoracolumbar longissimus muscle were taken for analysis. pH value of muscle at the fifth rib location was measured using a Mettler MP120 pH meter (Zurich, Switzerland), while color traits (lightness (L*), redness (a*) and yellowness (b*)) were evaluated with a Minolta CR-10 handheld colorimeter (Konica Minolta Optics, Osaka, Japan) based on the CIELAB color system [14].

#### 2.2.4. Histological Examination

Intestinal morphology was analyzed according to the previous method [15,16]. Briefly, samples were fixed, paraffin-embedded, and sectioned into 5 μm slices for hematoxylin and eosin (H&E) staining. Morphometric measurements included villus height (tip to crypt opening) and crypt depth (crypt mouth to the base). For each sample, ten intact villus-crypt structures per slide were examined across five slides at 40× magnification, with image analysis conducted using ImageJ software (Version 1.38, National Institute of Mental Health, Bethesda, MD, USA).

#### 2.2.5. Antioxidant Parameters, Diamine Oxidase (DAO) and D-Lactic Acid Levels

The contents of malondialdehyde (MDA), catalase (CAT), total antioxidant capacity (T-AOC), GSH-PX, total superoxide dismutase (T-SOD), D-lactate (D-LA) and diamine oxidase (DAO) in serum or liver were measured by corresponding assay kits (Nanjing Jiancheng Institute of Bioengineering, Nanjing, China) according to the manufacturer’s instructions.

#### 2.2.6. Analysis of Serum Indexes

Plasma glucose (GLU), triglyceride (TG), total protein (TP), albumin (ALB), carbamide (UREA), total cholesterol (TCHO), high density lipoprotein (HDL) and low density lipoprotein (LDL) concentrations were measured using an Automatic Biochemical Analyzer (Hitachi 7170A, Tokyo, Japan) with corresponding kits commercially available from Hitachi (Japan).

#### 2.2.7. Statistical Analysis

All data were analyzed using the general statistical linear model (GLM) procedure of SAS 9.0 software (SAS Inst. Inc., Cary, NC, USA). For production performance analysis, *n* = 8/treatment; for slaughtering performance, meat quality, serum indexes, antioxidant capacity, intestinal morphology and intestinal permeability, *n* = 8. Differences among treatments were examined using Duncan’s multiple range test and were considered to be significant at *p* < 0.05. The values are expressed as the mean ± SEM.

## 3. Results

### 3.1. Effects of Dietary Tributyrin Addition on the Production Performance and Slaughter Performance of Rabbits

**Figure 1:**
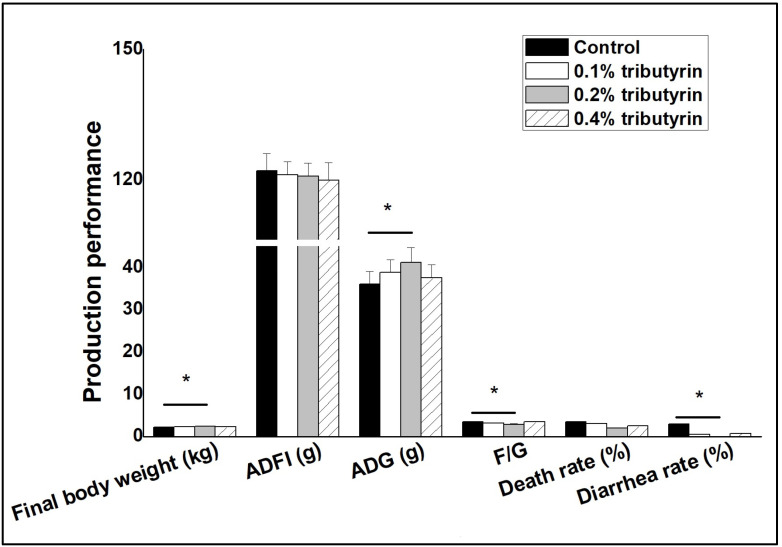
Effects of dietary tributyrin addition on production performance of rabbits. * indicates significant differences (*p* < 0.05). Production performance includes final body weight, ADFI, ADG, F/G, death rate and diarrhea rate.

Dietary supplementation with tributyrin did not significantly affect ADFI and death rate (*p* > 0.05, Figure 1). Compared with control, dietary addition of 0.2% tributyrin significantly increased FBW and ADG, but significantly decreased F/G (*p* < 0.05). The effect of dietary tributyrin addition on rabbit slaughter performance is shown in Table 2. Tributyrin supplementation in the diet did not significantly affect full-bore rate and half-bore rate (*p* > 0.05). As well, dietary tributyrin supplementation did not significantly affect the weight and index of spleen and liver (*p* > 0.05). Dietary addition of 0.1% and 0.2% tributyrin significantly increased the full-bore weight and the half-bore weight (*p* < 0.05).

### 3.2. Effects of Dietary Tributyrin Addition on Meat Quality and Serum Indexes of Rabbits

As shown in Table 3 and Table 4, dietary addition of 0.1% tributyrin significantly decreased pH value of muscle in 45 min after slaughter compared with control (*p* < 0.05, Table 3), while dietary supplementation with tributyrin did not significantly affect the L*, a*, b* or pH value of muscle in 24 h after slaughter (*p* > 0.05). Compared with control, dietary addition of 0.2% tributyrin significantly increased the serum glucose level (*p* < 0.05, Table 4). Compared with control, dietary addition of tributyrin did not significantly affect the levels of serum TP, ALB, UREA, TCHO, TG, HDL and LDL.

### 3.3. Effects of Dietary Tributyrin Addition on Antioxidant Capacity of Rabbits

As shown in Table 5, the content of GSH-PX in serum was increased in rabbits fed the diet with 0.1–0.2% tributyrin supplementation (*p* < 0.05). Compared with control, dietary addition of tributyrin significantly increased the content of T-SOD in serum (*p* < 0.01), but did not significantly affect the content of MDA, CAT and T-AOC in serum.

As shown in Table 6, the content of GSH-PX in the liver was increased in rabbits fed the diets with 0.1–0.2% tributyrin supplementation (*p* < 0.05). Compared with control, dietary addition of 0.2% tributyrin significantly increased the content of T-SOD in the liver (*p* < 0.05). Compared with control, dietary addition of tributyrindid did not significantly affect the content of MDA, CAT and T-AOC in the liver.

### 3.4. Effects of Dietary Tributyrin Addition on the Intestinal Morphology and Intestinal Permeability of Rabbits

As shown in Table 7, dietary addition of 0.2% tributyrin significantly increased the villus height and the ratio of villus height to crypt depth (V/C) in the duodenum (*p* < 0.05). Dietary addition of 0.1–0.2% tributyrin significantly decreased the crypt depth (*p* < 0.05) in the duodenum. In the jejunum, dietary addition of tributyrin (0.1–0.4%) significantly increased the V/C (*p* < 0.05), but significantly decreased crypt depth (*p* < 0.05). Compared with control, dietary addition of tributyrin did not significantly affect the villus height in the jejunum and ileum (*p* > 0.05). Dietary addition of tributyrin (0.1–0.4%) significantly decreased crypt depth in the ileum (*p* < 0.05). Dietary addition of 0.1–0.2% tributyrin significantly increased the V/C in the ileum (*p* < 0.05). Dietary addition of tributyrin (0.1–0.4%) significantly decreased DAO level in serum (*p* < 0.05, Table 8). Although dietary addition of 0.4% tributyrin did not significantly affect the D-lactase level in serum, dietary addition of 0.1–0.2% tributyrin significantly decreased the D-lactase level in serum (*p* < 0.05).

## 4. Discussion

### 4.1. Dietary Tributyrin Addition Improved Production Performance of Rabbits

In recent years, tributyrin has been increasingly utilized as a dietary supplement to enhance the health and growth performance of herbivorous livestock. Tributyrin supplementation increased the growth performance of weaned dairy calves and weaned lambs [17]. By acidifying the feed, tributyrin can potentially inhibit fungal growth, thereby reducing the production of mycotoxins in the feed, which reduces intestinal pressure and improves feed efficiency [18]. Our findings indicate that dietary 0.2% tributyrin supplementation significantly boosts growth performance in weaned rabbits, as demonstrated by elevated ADG, increased carcass weight and enhanced feed utilization. This improvement is likely mediated through tributyrin’s stimulation of gastrointestinal tract (GIT) development. The rabbit’s digestive system is uniquely structured, and its maturation directly influences nutrient absorption and growth efficiency. Our results are consistent with the previous findings [19], indicating that tributyrin supplementation can improve growth performance by improving the intestinal health of weaned piglets and broilers [14,20].

### 4.2. Dietary Tributyrin Addition Improved Meat Quality of Rabbits

Many studies have found that tributyrin can improve the meat quality in some mammals and poultry, but there are no relevant studies in rabbits. Chen et al. [21] found that dietary addition of 0.04–0.32% tributyrin did not significantly alter the pH value and meat color of muscle in 45 min or 24 h after slaughter of broilers. But the addition of 0.16% tributyrin improved the meat quality of skeletal muscle of broilers by reducing the dripping loss and shear force. Wang et al. [22] found that supplementing 0.2% tributyrin could improve the meat quality including muscle pH value and a* value in 45 min or 24 h after slaughter in weaned lambs by modifying the amino acid and fatty acid levels. The present study showed that the addition of 0.1% tributyrin decreased the pH value of muscle 45 min after slaughter, but did not affect the meat color of rabbit muscle. These results suggest that the effect of tributyrin on meat quality may be species-specific. The action dose and the target effect of tributyrin are different in different animals. pH value is an important indicator of meat quality. The decomposition of proteins by microorganisms can produce alkaline substances, leading to an increase in pH value. Decreased pH value after tributyrin treatment indicates that tributyrin can increase the freshness of rabbit meat and extend its shelf life.

### 4.3. Dietary Tributyrin Addition Improved the Antioxidant Ability of Rabbits

Under normal physiological conditions, a dynamic balance exists between oxidative processes and antioxidant defense mechanisms [23]. However, weaning stress disrupts this homeostasis, leading to oxidative stress through an imbalance between oxidants and antioxidants [24]. CAT, GSH-PX and T-SOD play a crucial role in the organism’s antioxidant system, which can protect cells from the damage caused by oxidative stress, maintain the stability of the intracellular environment and reflect the organism’s health status and antioxidant capacity [25]. In our study, dietary addition of 0.1–0.2% tributyrin increased the content of GSH-PX and T-SOD in serum and liver in rabbits, certifying that tributyrin exhibits an alleviation effect on weaning stress-induced oxidative stress in rabbits, partly by improving the antioxidant capacity. These results are in agreement with swimming crab and broilers [26,27]. Peng et al. [26] found that tributyrin could relieve hepatopancreas oxidative stress by enhancing cellular antioxidant capacity.

### 4.4. Dietary Tributyrin Addition Improved Intestinal Health of Rabbits

Butyrate, produced by bacterial fermentation in the rabbit cecum, has anti-cancer and anti-inflammatory effects and has a positive effect on the rabbit intestine [28]. Tributyrin, as a precursor of butyric acid, contains three butyric acid molecules, which can be directly released into the gut and improve intestinal health and intestinal development [29]. The increased serum glucose level in rabbits fed 0.2% tributyrin reflects a sufficient energy supply state. Moreover, tributyrin can increase the mRNA levels of glucose transport protein-1 and glucose transporter-2 in the jejunum and promote the rapid absorption of glucose in the intestine [30]. In addition, the large amount of butyric acid from tributyrin decomposition can directly supply energy to intestinal epithelial cells, which reduces the glucose consumption during the process of entering the blood. The intestinal mucosal barrier plays an important role in nutrient transportation. The increased V/C facilitates the absorption of nutrients by the intestine [31]. The previous study found that tributyrin can improve the intestinal morphology in piglets and broilers [32,33], which is in line with our present study, indicating that dietary addition of 0.1–0.4% tributyrin increased the V/C in duodenum, jejunum and ileum in weaned rabbits. These results suggest that tributyrin can accelerate the regeneration of intestinal epithelial cells [27], improve the intestinal mucosal barrier and increase the absorption capacity and nutrient digestibility in the small intestine [34], which improves the feed conversion efficiency of rabbits fed tributyrin.

Under normal conditions, DAO is mainly produced in the small intestine and is scarcely present in blood [35]. Furthermore, D-lactic acid, a bacterial fermentation product, predominantly localizes in the intestinal lumen and translocates into systemic circulation during gut barrier dysfunction [36]. Consequently, both serum DAO and D-lactic acid are considered reliable biomarkers for evaluating intestinal mucosal barrier integrity [37,38]. A study in rabbits demonstrates that weaning stress significantly compromises small intestinal barrier function [39]. Our findings reveal that tributyrin supplementation effectively reduces circulating levels of D-lactic acid and DAO in weaned rabbits, suggesting its protective role in maintaining intestinal barrier integrity, decreasing gut permeability and lowering diarrhea incidence.

## 5. Conclusions

In conclusion, dietary addition of tributyrin could improve the production performance and antioxidant ability of liver and serum, and improve the intestinal health by decreasing the diarrhea rate and the intestinal permeability and improving intestinal morphology in weaned rabbits. Based on the present results, the optimal level of tributyrin is 0.2% in the weaned rabbit diet.

## Figures and Tables

**Table 1:** Composition and nutrient levels of basal diets (air-dry basis).

Items	Content
Ingredients	
Corn	10.00
Soybean meal	7.00
Corn germ meal	8.00
Wheat bran	29.00
Husk powder	10.00
Sunflower meal	7.00
Alfalfa	8.20
Peanut shell	10.00
Middling	5.00
Soybean oil	0.80
Premix ^(1)^	5.00
Total	100.00
Measured chemical composition	
Digestive energy (MJ/kg)	10.21
Dry matter (%)	88.31
Crude protein (%)	15.97
Ash (%)	5.73
Ether extract (%)	3.43
Crude fiber (%)	16.49
Calcium (%)	0.31
Phosphorus (%)	0.53

^(1)^ The premix provides per kilogram of feed: Vitamin A, 8000 IU; Vitamin D3, 1000 IU; Vitamin E, 50 mg; Vitamin K3, 2.3 mg; Vitamin B1, 1.75 mg; Vitamin B2, 6.9 mg; Vitamin B3, 28.45 mg; Vitamin B5, 6.7 mg; Vitamin B9, 0.6 mg; Vitamin B12, 2.2 mg; Choline, 420 mg; Lysine, 1500 mg; Methionine, 1500 mg; Copper, 30 mg; Iron, 100 mg; Manganese, 30 mg; Magnesium, 150 mg; Iodine, 0.1 mg.

**Table 2:** Effects of dietary tributyrin addition on slaughtering performance of rabbits.

Item	Tributyrin Level (%)				SEM	*p* Value
	0	0.1	0.2	0.4		
Full-bore weight, kg	1.19 ^b^	1.31 ^a^	1.32 ^a^	1.22 ^b^	0.10	0.0280
Half-bore weight, kg	1.28 ^c^	1.39 ^a,b^	1.41 ^a^	1.30 ^b,c^	0.11	0.0272
Full-bore rate, %	55.37	56.85	56.26	56.32	2.75	0.7030
Half-bore rate, %	59.56	60.74	60.32	60.20	2.80	0.8317
Spleen weight, g	2.00	1.93	2.36	1.93	0.70	0.4638
Spleen index, g/kg	0.94	0.84	1.00	0.90	0.28	0.6570
Liver weight, g	67.88	72.88	65.76	65.19	12.14	0.4900
Liver index, g/kg	31.85	31.78	28.37	30.09	5.39	0.4251

Note: in the superscript of the same row, different letters indicate significant differences (*p* < 0.05), while no letters or the same letters indicate no significant differences (*p* > 0.05).

**Table 3:** Effects of dietary tributyrin addition on meat quality of rabbits.

Item	Tributyrin Level (%)				SEM	*p* Value
	0	0.1	0.2	0.4		
L*45 min	32.09	32.91	32.65	31.14	2.05	0.2365
a*45 min	33.34	32.07	32.43	32.58	1.79	0.4789
b*45 min	−3.07	−2.94	−3.06	−3.06	0.44	0.9071
pH 45 min	6.76 ^a^	6.65 ^b^	6.72 ^a^	6.69 ^a,b^	0.07	0.0175
L*24 h	42.77	42.78	41.74	43.57	3.12	0.6377
a*24 h	40.52	40.77	39.22	41.07	2.83	0.4834
b*24 h	1.13	1.72	1.370	1.72	2.10	0.9100
pH 24 h	5.80	5.77	5.79	5.77	0.06	0.6677

Note: in the superscript of the same row, different letters indicate significant differences (*p* < 0.05), while no letters or the same letters indicate no significant differences (*p* > 0.05).

**Table 4:** Effects of dietary tributyrin addition on serum indexes of rabbits.

Item	Tributyrin Level (%)				SEM	*p* Value
	0	0.1	0.2	0.4		
TP (g/L)	62.18	69.11	68.78	62.83	1.03	0.5441
ALB (g/dL)	35.12	40.20	38.42	36.25	4.40	0.1400
Glucose (mmol/L)	7.40 ^b^	7.79 ^a,b^	8.187 ^a^	7.70 ^a,b^	0.58	0.0206
UREA (mmo/L)	7.76	7.96	8.64	7.91	0.94	0.4219
TCHO (g/L)	1.63	1.40	1.77	1.59	0.46	0.3930
TG (mmol/L)	0.964 ^a,b^	1.259 ^a^	0.884 ^b^	0.788 ^b^	0.32	0.0094
HDL (mmol/L)	0.63	0.65	0.67	0.58	0.15	0.6396
LDL (mmol/L)	0.43	0.46	0.53	0.45	0.17	0.6420

Note: in the superscript of the same row, different letters indicate significant differences (*p* < 0.05), while no letters or the same letters indicate no significant differences (*p* > 0.05).

**Table 5:** Effects of dietary tributyrin addition on serum antioxidant indexes of rabbits.

Item	Tributyrin Level (%)				SEM	*p* Value
	0	0.1	0.2	0.4		
MDA (nM/mL)	2.74	0.88	2.47	2.14	1.50	0.2690
CAT (U/mL)	2.06	2.91	2.70	2.43	0.93	0.8455
T-AOC (mM/mL)	0.99	0.79	0.88	0.87	1.37	0.2577
GSH-PX (U/mL)	126.78 ^b^	151.06 ^a^	155.03 ^a^	131.65 ^a,b^	20.03	0.0043
T-SOD (U/mL)	31.00 ^c^	35.88 ^b^	40.02 ^a^	34.10 ^b^	1.90	0.0021

Note: in the superscript of the same row, different letters indicate significant differences (*p* < 0.05), while no letters or the same letters indicate no significant differences (*p* > 0.05).

**Table 6:** Effects of dietary tributyrin addition on liver antioxidant indexes of rabbits.

Item	Tributyrin Level (%)				SEM	*p* Value
	0	0.1	0.2	0.4		
MDA (nmol/mgprot)	1.00	1.54	2.26	1.81	1.46	0.5294
CAT (U/mgprot)	52.70	50.19	50.96	53.72	7.09	0.8364
T-AOC (U/mgprot)	0.47	0.71	0.74	0.51	0.312	0.4488
GSH-PX (U/mgprot)	40.36 ^b^	53.21 ^a^	59.90 ^a^	40.72 ^b^	10.35	0.0027
T-SOD (U/mgprot)	0.09 ^b^	0.10 ^a,b^	0.11 ^a^	0.09 ^b^	0.01	0.0346

Note: in the superscript of the same row, different letters indicate significant differences (*p* < 0.05), while no letters or the same letters indicate no significant differences (*p* > 0.05).

**Table 7:** Effects of dietary tributyrin addition on the intestinal morphology of rabbits.

Seat	Item	Tributyrin Level (%)				SEM	*p* Value
		0	0.1	0.2	0.4		
Duodenum	Villus height (μm)	739.50 ^b^	818.00 ^a,b^	873.33 ^a^	787.60 ^b^	61.81	0.0012
	Crypt depth (μm)	457.25 ^a^	321.75 ^b^	222.50 ^c^	399.00 ^a,b^	92.22	0.0011
	V/C	1.95 ^b^	2.60 ^b^	3.44 ^a^	2.22 ^b^	0.73	0.0051
Jejunum	Villus height (μm)	634.00	680.40	791.25	702.33	121.90	0.3657
	Crypt depth (μm)	485.33 ^a^	264.4 ^b^	182.25 ^b^	268.33 ^b^	107.25	0.0007
	V/C	1.33 ^c^	2.69 ^b^	4.57 ^a^	2.63 ^b^	1.26	0.0081
Ileum	Villus height (μm)	599.25	684.75	728.25	617.50	91.78	0.2055
	Crypt depth (μm)	303.00 ^a^	220.50 ^b^	197.50 ^b^	233.50 ^b^	45.74	0.0201
	V/C	2.06 ^c^	3.13 ^a,b^	3.81 ^a^	2.60 ^b,c^	0.62	0.0017

Note: In the superscript of the same row, different letters indicate significant differences (*p* < 0.05), while no letters or the same letters indicate no significant differences (*p* > 0.05).

**Table 8:** Effects of dietary tributyrin addition on the intestinal permeability of rabbits.

Item	Tributyrin Level (%)				MSE	*p* Value
	0	0.1	0.2	0.4		
DAO (μg/mL)	220.55 ^a^	175.42 ^b^	159.10 ^b^	187.39 ^a,b^	33.46	0.0228
D-lactic acid (μg/mL)	959.90 ^a^	768.24 ^b^	709.41 ^b^	721.66 ^b^	137.08	0.0046

Note: In the superscript of the same row, different letters indicate significant differences (*p* < 0.05), while no letters or the same letters indicate no significant differences (*p* > 0.05).

## Data Availability

The data supporting this study’s findings are available upon request from the corresponding author.
